# Quantifying Mg^2+^ Binding to ssDNA Oligomers: A Self-Consistent Field Theory Study at Varying Ionic Strengths and Grafting Densities

**DOI:** 10.3390/polym10121403

**Published:** 2018-12-18

**Authors:** Merina Jahan, Mark J. Uline

**Affiliations:** 1 Department of Chemical Engineering, University of South Carolina, Columbia, SC 29208, USA; mjahan@email.sc.edu; 2 Biomedical Engineering Program, University of South Carolina, Columbia, SC 29208, USA

**Keywords:** aptamers, self-consistent field theory, polyelectrolytes, metal binding, molecular modeling, single-stranded DNA (ssDNA)

## Abstract

The performance of aptamer-based biosensors is crucially impacted by their interactions with physiological metal ions, which can alter their structures and chemical properties. Therefore, elucidating the nature of these interactions carries the utmost importance in the robust design of highly efficient biosensors. We investigated Mg${}^{2+}$ binding to varying sequences of polymers to capture the effects of ionic strength and grafting density on ion binding and molecular reorganization of the polymer layer. The polymers are modeled as ssDNA aptamers using a self-consistent field theory, which accounts for non-covalent ion binding by integrating experimentally-derived binding constants. Our model captures the typical polyelectrolyte behavior of chain collapse with increased ionic strength for the ssDNA chains at low grafting density and exhibits the well-known re-entrant phenomena of stretched chains with increased ionic strength at high grafting density. The binding results suggest that electrostatic attraction between the monomers and Mg${}^{2+}$ plays the dominant role in defining the ion cloud around the ssDNA chains and generates a nearly-uniform ion distribution along the chains containing varying monomer sequences. These findings are in qualitative agreement with recent experimental results for Mg${}^{2+}$ binding to surface-bound ssDNA.

## 1. Introduction

Aptamers are an important class of biomolecules consisting of single-stranded DNA (ssDNA), RNA, or peptides that can fold into unique secondary and tertiary structures for shape-specific target recognition [1]. Due to the highly specific and selective nature of their target binding, aptamers are widely studied for a range of applications from biosensing [2,3] to drug design [4,5,6]. A recent work reported a major breakthrough in biosensor research by using aptamers with field-effect transistors to overcome the Debye length limitations [7]. Aptamers are polyelectrolytic in nature with their monomer units (nucleobases) participating in acid-base equilibrium and counterion binding reactions with the surrounding solution environments. Charge regulation and counterion binding in aptamers, or polyelectrolytes in general, are modulated by the metal ions present in the system, which can non-trivially alter their chemical and structural properties [8,9,10,11,12,13]. The presence of metal ions affects the performance of the aptamers as biosensing probes or therapeutics [14,15,16] due to the electrostatic screening of the charges on their surface, which changes their structure and chemistry. These interactions of aptamers with metal ions are complicated in nature owing to the fact that multiple binding sites on the nucleobases are capable of such interactions, following different binding pathways and thus having varying energy landscapes [17,18]. In this work, we have particularly addressed magnesium ion (Mg${}^{2+}$) binding because of its relevance to almost all nucleic acid-related biological processes in the intracellular environment [19,20,21].

A myriad of computational studies has been conducted with Molecular Dynamics (MD) [22,23,24,25,26,27,28,29,30] and Monte Carlo (MC) [31] simulations to elucidate the nature of metal ion binding to nucleic acids. Most of such theoretical studies are based on double-stranded DNA (dsDNA)–monovalent cation (such as Na${}^{+}$, K${}^{+}$) interactions [25,27,29,30]. Among the few that include multivalent cations, Hayes et al. [26] employed a hybrid structure-based MD model to count explicitly the number of excess Mg${}^{2+}$ ions bound to RNA sequences in the presence of background potassium chloride with a Manning condensation estimated by a non-linear Poisson–Boltzmann equation. Li et al. [28] used implicit Mg${}^{2+}$ binding to dsDNA sequences with classical MD simulation to study the effect of counterion condensation on DNA structure and conformational dynamics. While atomistic MD simulations give a full distribution of the ion atmosphere around the nucleic acids, they suffer from drawbacks due to the enormous computational cost and the choice of force fields that might lead to over- or under-estimation of the same ion cloud [27,32]. These studies also rely heavily on parameterization to match experimental studies, which imposes unrestrained bias toward their agreement with experimental results [32]. On the other hand, almost all theoretical studies consider nucleic acid chains in bulk conditions; therefore, characteristics of nucleic acid strands end-tethered to a surface remain elusive.

Along this line, we address metal ion binding to surface-anchored nucleic acid oligomers with a Self-Consistent Field Theory (SCFT) approach to construct a comprehensive and statistically-robust model for quantifying the number of Mg${}^{2+}$ ions bound to each chain, while capturing the ion-binding effect on their structure and properties. The molecular model analyzes Mg${}^{2+}$ binding to nucleic acid oligomers containing adenine (A) and guanine (G) nucleobases, while capturing the details of experimental studies for similar systems as precisely as possible. Metal ion binding to the monomers is explicitly included with equilibrium binding reactions by using experimentally-derived [33] binding free energies for relevant binding modes [34]. The molecular model characterizes the spatial variation of the structure and properties of the oligonucleotide chains along the distance from the grafting surface, at varying ionic strength and grafting densities, and quantifies the number of bound ions at thermodynamic equilibrium with the oligonucleotides. The model explicitly accounts for the thermodynamic, structural, and electrostatic properties of all the species involved in the system, while remaining free of adjustable parameters. Despite the many benefits of using an SCFT approach to studying metallic interactions with oligonucleotides, there are several drawbacks. The first is that a form of the mean-field approximation must be used to model the intermolecular interactions. While much of the relevant physics related to correlations is present in our model, not all of the correlations are completely captured. As discussed in much greater detail later, this calculation also uses a model for building the configurational space of the oligonucleotides that could possibly be better represented by using simulation results, and we are performing a one-dimensional calculation that only treats inhomogeneities in the direction normal to the surface. Lateral heterogeneity would have to be accounted for in situations where there are appreciable defects in the grafting of the oligonucleotides to the surface, or if there is a need to capture microphase separation, where the system would be able to adopt a periodic two-dimensional structure. This field theoretic model helps to set up the foundation for future studies involving secondary and tertiary structures of aptamers interacting with multivalent metal ions.

## 2. Theoretical Methodology

**Figure 1:**
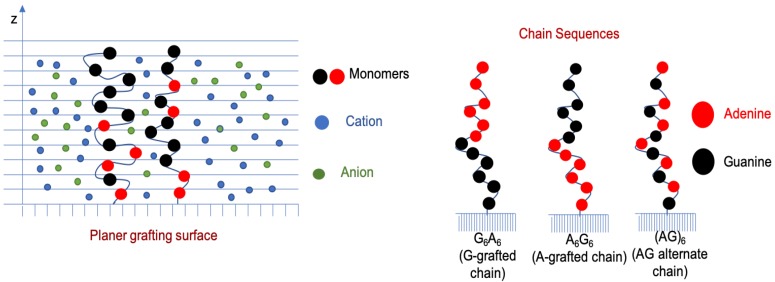
Schematic representation of an end-grafted polymer in a salt solution environment (**left**) and chain sequences used for molecular modeling (**right**).

The theoretical model in this work is developed to represent surface-grafted ssDNA oligomers as a co-polymer with two monomer units, adenine (A) and guanine (G), at a coarse-grained level. We explicitly consider the physical and chemical properties of these nucleobases in a solution environment to capture their behavior as accurately as possible. We study three chain sequences: the diblock co-polymer of A and G with the A-end grafted to the surface (A${}_{6}$G${}_{6}$, a block of six adenine monomers followed by a block of six guanine monomers), the diblock of A and G with the G-end grafted to the surface (G${}_{6}$A${}_{6}$, a block of six guanine monomers followed by a block of six adenine monomers), and an alternating sequence of A and G ((AG)${}_{6}$, one adenine monomer followed by a guanine monomer in an alternating manner) (Figure 1). Each of these chains contains 12 monomers at varying grafting densities and MgCl${}_{2}$ concentrations, while keeping the solution temperature and background NaCl concentration fixed at 298 K and 10 mM, respectively. NaCl is added to the system to comply with the relevant experimental study by Holland et al. [34], which hypothesizes that Mg${}^{2+}$ might replace Na${}^{+}$ in a probable binding pathway.

The theoretical model is constructed using a Self-Consistent Field Theory (SCFT) approach for a single polyelectrolyte chain in a field of interacting species [8,35,36,37]. The polyelectrolyte chains are end-tethered to a surface and submerged in a salt and water bath, containing both NaCl and MgCl${}_{2}$ salts. The motivation behind our study is to discover the extent of Mg${}^{2+}$ ion binding to the polyelectrolytes and how it changes the structure and properties of the polyelectrolyte chains. In this molecular model, $N_{p}$ polyelectrolyte chains are end-grafted to a surface with cross-sectional area *A*. We assume the system to be homogeneous in the *x* and *y* directions and heterogeneous in the *z* direction, along the distance perpendicular to the grafting surface. Within the field theory framework, the heterogeneity normal to the surface is accounted for by discretizing the system space into a number of layers. The concentrations of the salts are converted to a density field to determine their individual contribution to the field, as a function of distance from the grafting surface. Cation binding to the polyelectrolytes is considered within the scope of reaction equilibrium calculations, rather than condensation near the charged monomers [38], with binding reactions relevant to the experimental study of a similar system [34]. We start constructing the model by calculating the total Helmholtz free energy of the system, which is given by,

(1) $$F=-TS_{conf}-TS_{mix}+F_{chem}+F_{elect}+E_{rep}$$

Here, $S_{conf}$ is the conformational entropy of the grafted polymer chains, and $S_{mix}$ is the mixing or translational entropy of all the free species: water (*w*), protons (H${}^{+}$), hydroxyl ions (OH${}^{-}$), cations (Na${}^{+}$, Mg${}^{2+}$), and anions (Cl${}^{-}$, OH${}^{-}$). $F_{chem}$ is the free energy associated with the equilibrium reactions that the monomers undergo in this system. We have explicitly considered three equilibrium reactions for each monomer: (1) protonation and deprotonation via acid-base equilibrium reactions, (2) Mg${}^{2+}$ binding, and (3) Na${}^{+}$ binding. This yields a total of six equilibrium reactions accounted for in our model:

(2) $$A^{-}+H^{+}⟺AH$$

(3) $$G^{-}+H^{+}⟺GH$$

(4) $$A^{-}+Mg^{2+}⟺AMg^{+}$$

(5) $$G^{-}+Mg^{2+}⟺GMg^{+}$$

(6) $$A^{-}+Na^{+}⟺ANa$$

(7) $$G^{-}+Na^{+}⟺GNa$$

$F_{elect}$ is the total electrostatic energy due to the charged species and $E_{rep}$ is the repulsive interaction experienced between all species due to steric hindrance. *T* is the temperature of the system, which is held constant at 298 K. Expansion of all the energy and entropy terms gives the total Helmholtz free energy of the system,

(8) $$\begin{array}{cc} \frac{\beta F}{A} & =\sigma_{p}\sum_{\alpha }P\left(\alpha \right)\ln P\left(\alpha \right)+\int \rho_{w}\left(z\right)\left(\ln\rho_{w}\left(z\right)v_{w}-1\right)dz\\ & +\int \rho_{H^{+}}\left(z\right)\left(\ln\rho_{H^{+}}\left(z\right)v_{w}-1+\beta \mu_{H^{+}}^{0}\right)dz\\ & +\int \rho_{OH^{-}}\left(z\right)\left(\ln\rho_{OH^{-}}\left(z\right)v_{w}-1+\beta \mu_{OH^{-}}^{0}\right)dz\\ & +\int \rho_{Na^{+}}\left(z\right)\left(\ln\rho_{Na^{+}}\left(z\right)v_{w}-1+\beta \mu_{Na^{+}}\right)dz\\ & +\int \rho_{Mg^{2+}}\left(z\right)\left(\ln\rho_{Mg^{2+}}\left(z\right)v_{w}-1+\beta \mu_{Mg^{2+}}\right)dz\\ & +\int \rho_{Cl^{-}}\left(z\right)\left(\ln\rho_{Cl^{-}}\left(z\right)v_{w}-1+\beta \mu_{Cl^{-}}\right)dz\\ & +\beta \int [\langle \rho_{q}\left(z\right)\rangle \psi \left(z\right)+\frac{1}{2}\epsilon_{w}{\left(\frac{d\psi (z)}{dz}\right)}^{2}]dz\\ & +\int \langle \rho_{A}\left(z\right)\rangle [f_{A^{-}}\left(z\right)\ln f_{A^{-}}\left(z\right)+f_{AH}\left(z\right)\ln f_{AH}\left(z\right)\\ & +f_{ANa}\left(z\right)\ln f_{ANa}\left(z\right)+f_{AMg^{+}}\left(z\right)\ln f_{AMg^{+}}\left(z\right)\\ & +f_{A^{-}}\left(z\right)\beta \mu_{A^{-}}^{0}+f_{AH}\left(z\right)\beta \mu_{AH}^{0}+f_{ANa}\left(z\right)\beta \mu_{ANa}^{0}+f_{AMg^{+}}\left(z\right)\beta \mu_{AMg^{+}}^{0}]dz\\ & +\int \langle \rho_{G}\left(z\right)\rangle [f_{G^{-}}\left(z\right)\ln f_{G^{-}}\left(z\right)+f_{GH}\left(z\right)\ln f_{GH}\left(z\right)+f_{GNa}\left(z\right)\ln f_{GNa}\left(z\right)\\ & +f_{GMg^{+}}\left(z\right)\ln f_{GMg^{+}}\left(z\right)+f_{G^{-}}\left(z\right)\beta \mu_{G^{-}}^{0}+f_{GH}\left(z\right)\beta \mu_{GH}^{0}\\ & +f_{GNa}\left(z\right)\beta \mu_{GNa}^{0}+f_{GMg^{+}}\left(z\right)\beta \mu_{GMg^{+}}^{0}]dz \end{array}$$

The first term in Equation (8) stands for the structural or conformational energy ($TS_{conf}$) of the polyelectrolyte chains, where P(α) is the probability of the polymer to be in a conformational state, α, and $\sigma_{p}=\frac{N_{p}}{A}$ is the grafting density on the surface. The second through seventh term represent the total mixing energy ($TS_{mix}$) of all the mobile species in the system. $\rho_{i}\left(z\right)$ is the position-dependent density of species *i*. ${\mu^{0}}_{i}$ and $v_{i}$ are the standard chemical potential and the volume of species *i*, respectively. The second term is the translational energy of the undissociated water molecules. The third and fourth terms represent the translational entropy of H${}^{+}$ and OH${}^{-}$ ions, respectively, from the dissociation of water. The fifth, sixth, and seventh terms describe the translational entropy of the anions and cations (Mg${}^{2+}$, Na${}^{+}$, and Cl${}^{-}$) produced by the dissociation of the salts (MgCl${}_{2}$ and NaCl). We assume that the salts in this system are completely dissociated. The eighth term represents the contribution of the electrostatic energy, $F_{elect}$. ψ(z) is the electrostatic potential, and $\epsilon_{w}$ is the dielectric constant of water. $\langle \rho_{q}\left(z\right)\rangle$ is the ensemble average charge density of the system, given by, 

(9) $$\begin{array}{cc} \langle \rho_{q}\left(z\right)\rangle & =\sum_{i=Na^{+},Mg^{2+},Cl^{-},H^{+},OH^{-}}ez_{i}\rho_{i}\left(z\right)-e\langle \rho_{A}\left(z\right)\rangle \left(f_{A^{-}}\left(z\right)-f_{AMg^{+}}\left(z\right)\right)\\ & -e\langle \rho_{G}\left(z\right)\rangle \left(f_{G^{-}}\left(z\right)-f_{GMg^{+}}\left(z\right)\right) \end{array}$$

Here, $f_{i}\left(z\right)$ is the fraction of a monomer in different states, such as deprotonated or charged (A${}^{-}$, G${}^{-}$), protonated (AH, GH) or cation-bound (AMg${}^{+}$, GMg${}^{+}$), etc., along the distance from the grafting surface.

The ninth and tenth terms are derived from the reaction equilibrium of Equations (2)–(5), respectively. The reaction equilibrium energies include the entropy of mixing between charged and uncharged groups, as well as the standard chemical potentials of these groups. Any volume change of the polyelectrolyte segments due to protonation and ion-binding is neglected. The system is in contact with a bath of anions, cations, protons, and hydroxyl ions and, therefore, best described by a semi-grand canonical ensemble. The proper thermodynamic potential (*W*) is given by,

(10) $$W=F-\sum_{\gamma =w,Na^{+},Mg^{2+},Cl^{-},H^{+},OH^{-}}\mu_{\gamma }N_{\gamma }$$

Here, $N_{\gamma }$ is the total number of molecules of species γ and $\mu_{\gamma }$ is the exchange chemical potential of those species.

### Extremization of the Free Energy

The free energy equation is extremized subjected to two constraints. One is the incompressibility constraint, originating from the repulsive interactions between different species,

(11) $$\langle \phi_{p}\left(z\right)\rangle +\phi_{w}\left(z\right)+\phi_{Na^{+}}\left(z\right)+\phi_{Mg^{2+}}\left(z\right)+\phi_{H^{+}}\left(z\right)+\phi_{OH^{-}}\left(z\right)+\phi_{Cl^{-}}\left(z\right)=1$$

Here, $\langle \phi_{p}\left(z\right)\rangle$ is the ensemble average polymer volume fraction, which is given by,

(12) $$\begin{array}{c} \langle \phi_{p}\left(z\right)\rangle =\langle \rho_{A}\left(z\right)\rangle \left(f_{A^{-}}\left(z\right)v_{A^{-}}+f_{AH}\left(z\right)v_{AH}+f_{ANa}\left(z\right)v_{ANa}+f_{AMg^{+}}\left(z\right)v_{AMg^{+}}\right)\\ +\langle \rho_{G}\left(z\right)\rangle \left(f_{G^{-}}\left(z\right)v_{G^{-}}+f_{GH}\left(z\right)v_{GH}+f_{GNa}\left(z\right)v_{GNa}+f_{GMg^{+}}\left(z\right)v_{GMg^{+}}\right) \end{array}$$

$\langle \rho_{i}\left(z\right)\rangle$ is the average number density of monomer *i* at position *z*, expressed as,

(13) $$\langle \rho_{i=A,G}\left(z\right)\rangle =\sigma_{p}\sum_{\alpha }P\left(\alpha \right)n_{i=A,G}\left(\alpha ;z\right)v_{i=A,G}$$

$\phi_{i}\left(z\right)$ is the volume fraction of free species *i* in the system as a function of the distance from the grafting surface and defined as $\phi_{i}\left(z\right)=\rho_{i}v_{i}$.

The second constraint arises from the fact that the total number of each species of monomer is fixed. Hence, monomer fractions of different derivative species sum to unity,

(14) $$f_{A^{-}}\left(z\right)+f_{AH}\left(z\right)+f_{ANa}\left(z\right)+f_{AMg^{+}}\left(z\right)=1$$

(15) $$f_{G^{-}}\left(z\right)+f_{GH}\left(z\right)+f_{GNa}\left(z\right)+f_{GMg^{+}}\left(z\right)=1$$

Under these two constraints, the total thermodynamic potential per unit area is given by,

(16) $$\begin{array}{cc} \frac{\beta W}{A} & =\frac{\beta F}{A}-\sum_{k=w,Cl^{-},OH^{-}}\beta \mu_{k}\int \rho_{k}\left(z\right)dz\\ & -\beta \mu_{H^{+}}\int [\rho_{H^{+}}\left(z\right)+f_{AH}\left(z\right)\langle \rho_{A}\left(z\right)\rangle \\ & +f_{GH}\left(z\right)\langle \rho_{G}\left(z\right)\rangle ]dz\\ & -\beta \mu_{Na^{+}}\int \left[\rho_{Na^{+}}\left(z\right)+f_{ANa}\left(z\right)\langle \rho_{A}\left(z\right)\rangle +f_{GNa}\left(z\right)\langle \rho_{G}\left(z\right)\rangle \right]dz\\ & -\beta \mu_{Mg^{2+}}\int \left[\rho_{Mg^{2+}}\left(z\right)+f_{AMg^{+}}\left(z\right)\langle \rho_{A}\left(z\right)\rangle +f_{GMg^{+}}\left(z\right)\langle \rho_{G}\left(z\right)\rangle \right]dz\\ & +\beta \int \lambda_{1}\left(z\right)\langle \rho_{A}\left(z\right)\rangle \left(f_{A^{-}}\left(z\right)+f_{AH}\left(z\right)+f_{ANa}\left(z\right)+f_{AMg^{+}}\left(z\right)-1\right)dz\\ & +\beta \int \lambda_{2}\left(z\right)\langle \rho_{G}\left(z\right)\rangle \left(f_{G^{-}}\left(z\right)+f_{GH}\left(z\right)+f_{GNa}\left(z\right)+f_{GMg^{+}}\left(z\right)-1\right)dz\\ & +\beta \int \pi \left(z\right)(\langle \phi_{p}\left(z\right)\rangle +\phi_{w}\left(z\right)+\phi_{Na^{+}}\left(z\right)+\phi_{Mg^{2+}}\left(z\right)+\phi_{H^{+}}\left(z\right)\\ & +\phi_{OH^{-}}\left(z\right)+\phi_{Cl^{-}}\left(z\right)-1)dz \end{array}$$

Here, π(z), $\lambda_{1}\left(z\right)$, and $\lambda_{2}\left(z\right)$ are Lagrange multipliers to incorporate the constraints into the free energy equation.

Extremization of the thermodynamic potential Equation (16), with respect to the relevant variables, gives the expressions for the equilibrium values of $\rho_{i}\left(z\right)$, $f_{i}\left(z\right)$, ψ(z), and P(α). From functional extremization, the density profiles of the free species are given by the following equations,

(17) $$\rho_{w}\left(z\right)w_{w}=\exp\left[-\beta \pi \left(z\right)v_{w}\right]$$

(18) $$\rho_{H^{+}}\left(z\right)v_{w}=\exp\left[\beta \mu_{H^{+}}-\beta \mu_{H^{+}}^{0}-\beta \pi \left(z\right)v_{H^{+}}-\beta \psi \left(z\right)e\right]$$

(19) $$\rho_{OH^{-}}\left(z\right)v_{w}=\exp\left[\beta \mu_{OH^{-}}-\beta \mu_{OH^{-}}^{0}-\beta \pi \left(z\right)v_{OH^{-}}+\beta \psi \left(z\right)e\right]$$

(20) $$\rho_{Na^{+}}\left(z\right)v_{w}=\exp\left[\beta \mu_{Na^{+}}-\beta \mu_{Na^{+}}^{0}-\beta \pi \left(z\right)v_{Na^{+}}-\beta \psi \left(z\right)e\right]$$

(21) $$\rho_{Mg^{2+}}\left(z\right)v_{w}=\exp\left[\beta \mu_{Mg^{2+}}-\beta \mu_{Mg^{2+}}^{0}-\beta \pi \left(z\right)v_{Mg^{2+}}-2\beta \psi \left(z\right)e\right]$$

(22) $$\rho_{Cl^{-}}\left(z\right)v_{w}=\exp\left[\beta \mu_{Cl^{-}}-\beta \mu_{Cl^{-}}^{0}-\beta \pi \left(z\right)v_{Cl^{-}}+\beta \psi \left(z\right)e\right]$$

Functional extremization with respect to the monomer fractions, $f_{i}\left(z\right)$, yields the governing equations for the chemical equilibrium reactions of both the monomers, A and G:

(23) $$\frac{f_{A^{-}}\left(z\right)}{f_{AH}\left(z\right)}=K_{AH}^{0}\frac{\exp\left(-\beta \pi \left(z\right)\Delta v_{AH}\right)}{\rho_{H^{+}}\left(z\right)v_{w}}$$

(24) $$\frac{f_{A^{-}}\left(z\right)}{f_{ANa}\left(z\right)}=K_{ANa}^{0}\frac{\exp\left(-\beta \pi \left(z\right)\Delta v_{ANa}\right)}{\rho_{Na^{+}}\left(z\right)v_{w}}$$

(25) $$\frac{f_{A^{-}}\left(z\right)}{f_{AMg^{+}}\left(z\right)}=K_{AMg^{+}}^{0}\frac{\exp\left(-\beta \pi \left(z\right)\Delta v_{AMg^{+}}\right)}{\rho_{Mg^{2+}}\left(z\right)v_{w}}$$

(26) $$\frac{f_{G^{-}}\left(z\right)}{f_{GH}\left(z\right)}=K_{GH}^{0}\frac{\exp\left(-\beta \pi \left(z\right)\Delta v_{GH}\right)}{\rho_{H^{+}}\left(z\right)v_{w}}$$

(27) $$\frac{f_{G^{-}}\left(z\right)}{f_{GNa}\left(z\right)}=K_{GNa}^{0}\frac{\exp\left(-\beta \pi \left(z\right)\Delta v_{GNa}\right)}{\rho_{Na^{+}}\left(z\right)v_{w}}$$

(28) $$\frac{f_{G^{-}}\left(z\right)}{f_{GMg^{+}}\left(z\right)}=K_{GMg^{+}}^{0}\frac{\exp\left(-\beta \pi \left(z\right)\Delta v_{GMg^{+}}\right)}{\rho_{Mg^{2+}}\left(z\right)v_{w}}$$

The quantity, $K_{i}^{0}=\exp\left(-\beta \Delta G_{i}^{0}\right)$, corresponds to the chemical equilibrium constant that is derived from the standard chemical free energy, $\Delta G_{i}^{0}$, of the respective formation reactions for AH, GH, ANa, GNa, $AMg^{+}$, or $GMg^{+}$. $\Delta v_{i}$ denotes the volume change due to the reactions. The change in the standard free energy for the reaction, $A^{-}+Mg^{2+}⟺AMg^{+}$, is given by: $\Delta G_{AMg^{+}}^{0}=\mu_{AMg^{+}}^{0}-\mu_{A^{-}}^{0}-\mu_{Mg^{2+}}^{0}$, and the volume change of reaction is $\Delta v_{AMg^{+}}=v_{AMg^{+}}-v_{A^{-}}-v_{Mg^{2+}}$. The reaction constants and the change in the volumes for the other reactions can be derived in a similar manner.

Extremization of the free energy with respect to the electrostatic potential yields the Poisson equation,

(29) $$\epsilon_{w}\frac{d^{2}\psi \left(z\right)}{dz^{2}}=-\langle \rho_{q}\left(z\right)\rangle$$

(30) $$\epsilon_{w}\frac{d\psi (z)}{dz}{|}_{z=0}=0,\lim_{r\to \infty }\psi \left(z\right)=0$$

The probability distribution function (pdf) is derived from the functional minimization with P(α),

(31) $$\begin{array}{cc} P(\alpha ) & =\frac{1}{e}\exp[-\int n_{A}\left(\alpha ;z\right)v_{A}\left(\ln f_{A^{-}}\left(z\right)+\beta \mu_{A^{-}}^{0}+\beta \pi \left(z\right)v_{A^{-}}-\beta e\psi \left(z\right)\right)dz\\ & -\int n_{G}\left(\alpha ;z\right)v_{G}\left(\ln f_{G^{-}}\left(z\right)+\beta \mu_{G^{-}}^{0}+\beta \pi \left(z\right)v_{G^{-}}-\beta e\psi \left(z\right)\right)]dz \end{array}$$

Equations (17)–(31) are solved simultaneously following the procedure described in the previous publications using this general approach [8,35,38]. These integro-differential equations are solved numerically by discretizing the space for a discretization length of 0.3 nm for 100 discrete layers. The solution of these sets of non-linear coupled equations yields the unknowns of the model, which are the Lagrange multiplier, π(z), and the electrostatic potential, ψ(z). The inputs necessary to solve the system of equations are the bulk concentrations of the salts, bulk pH, grafting density, volumes of different species, a set of polymer conformations, and the equilibrium reaction constants. The equilibrium constants for the binding reactions between the monomers (adenine and guanine) and the Mg${}^{2+}$ cations are obtained from the binding free energies of the Second Harmonic Generation (SHG) and Atomic Force Microscopy (AFM) studies of Holland et al. [34], by using the previously-mentioned equation for equilibrium constant, $K_{i}^{0}$. The binding free energies are −32.1 KJ/mol and −35.6 KJ/mol for adenine and guanine, respectively. The pKa values for G and A are 1.6 and 3.5, respectively [39]. The volume of Mg${}_{aq}^{2+}$ is 0.18 nm${}^{3}$, and the volumes for Na${}_{aq}^{+}$ and Cl${}_{aq}^{-}$ are 0.05 nm${}^{3}$. We did not include a change in volume upon binding in this analysis. The set of polymer conformations is derived using a Rotational Isomeric State (RIS) model [40]. The RIS model enumerates the configurations by changing the dihedral angles between connected monomers along the chain. There are three possible configurations: trans, or gauche-plus, or gauche-minus. We then use solid body rotations of each finished configuration to provide us with several million physical configurations to use in the calculation.

## 3. Results and Discussions

To elucidate the effects of Mg${}^{2+}$ binding on the structure and properties of a short chain surface-grafted A-G oligomer, we studied three different chain sequences: A-G diblock with the A-end grafted to the surface (A${}_{6}$G${}_{6}$), A-G diblock with the G-end grafted to the surface (G${}_{6}$A${}_{6}$), and A-G co-polymer with alternating A and G along the chain ((AG)${}_{6}$) (referring to Figure 1). We studied the variation of the MgCl${}_{2}$ concentration and the polymer surface coverage for the same chain types, as well as quantifying the number of bound Mg${}^{2+}$ ions to each chain in different solution conditions, while keeping the background NaCl concentration and pH constant, at 10 mM and 7.0, respectively. We present the quantitative results for all the chain sequences, and the qualitative results for the A-grafted chains only, as the results for the other chain systems do not deviate quantitatively from the A-grafted system. Our results provide valuable insight into the molecular details of this polyelectrolyte system in various biologically-relevant environments.

### 3.1. Effect of Sequence Heterogeneity on Mg${}^{2+}$ Binding

The number of bound Mg${}^{2+}$ per chain is calculated from the volume fraction of the polymer and the fraction of the Mg${}^{2+}$-bound monomers by using the following equation:

(32) $$N_{Mg^{2+}}/chain=\frac{\int \langle \phi_{p}\left(z\right)\rangle f_{PMg^{+}}\left(z\right)dz}{\sigma_{p}v_{p}}$$

**Figure 2:**
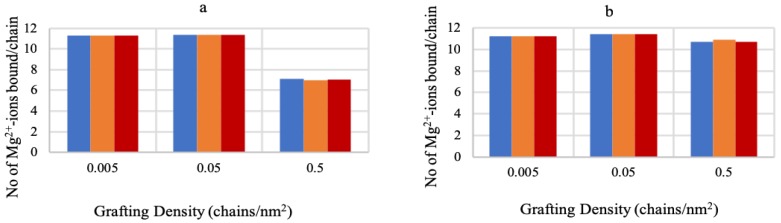
No. of bound Mg${}^{2+}$ to different ssDNA sequences at varying grafting densities for (**a**) 3 mM MgCl${}_{2}$ and (**b**) 180 mM MgCl${}_{2}$. The color bars correspond to the A-grafted chain (blue), G-grafted chain (yellow), and A-G alternate chain (red).

Figure 2a,b shows the number of bound Mg${}^{2+}$ with A${}_{6}$G${}_{6}$, G${}_{6}$A${}_{6}$ and (AG)${}_{6}$ sequences at different grafting densities for 3 mM and 180 mM MgCl${}_{2}$ content. The data in both plots demonstrate that, despite the sequence heterogeneity and variation on the grafting ends, all three sequences bind similar amounts of Mg${}^{2+}$ ions at different grafting densities and salt concentrations, except for the 0.5 chains/nm${}^{2}$ and 3 mM condition. The relatively low cation binding at this condition can be attributed to the unavailability of enough Mg${}^{2+}$ ions at the low concentration of the MgCl${}_{2}$ salt and high grafting density of the polymer chains. These results are in agreement with the experimental findings that Mg${}^{2+}$ ions do not aggregate around the strongest binder (guanine), and are uniformly distributed throughout the length of the chains [33]. This agrees with the notion that non-specific interactions due to the electrostatics of the sugar-phosphate backbone dominating the ion binding to the ssDNA chains, as the non-specific part of the free energy for adenine and guanine (−21 KJ/mol, from Holland et al. [34]) is much higher than the specific part of the free energies (−11.1 KJ/mol and −14.6 KJ/mol, respectively, from Holland et al. [34]), and the specific free energies are all within a few KJ of each other, hence making the ion binding less distinctive.

### 3.2. Effect of Ionic Strength and Grafting Density

**Figure 3:**
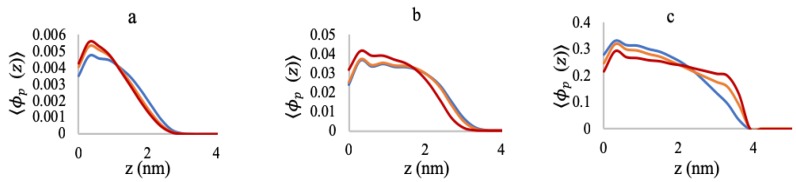
Total polymer volume fraction profiles as a function of distance from the grafting surface at (**a**) 0.005 chains/nm${}^{2}$, (**b**) 0.05 chains/nm${}^{2}$, and (**c**) 0.5 chains/nm${}^{2}$. Blue lines correspond to 3 mM MgCl${}_{2}$, yellow lines to 50 mM MgCl${}_{2}$, and red lines to 180 mM MgCl${}_{2}$.

Figure 3a–c shows the volume fraction profiles of the A-grafted chains (A${}_{6}$G${}_{6}$), along the distance from the grafting surface for low (0.005 chains/nm${}^{2}$), medium (0.05 chains/nm${}^{2}$), and high (0.5 chains/nm${}^{2}$) grafting densities, for a range of MgCl${}_{2}$ concentrations. At low grafting density (Figure 3a), as we increase the MgCl${}_{2}$ salt concentration from 3 mM–180 mM, the chain structures contract towards the grafting surface, and we get a distinct peak of the highest volume fractions at a distance close to the surface. This change in the chain structure is due to the reduction in negative charge of the polyelectrolytes by binding to the Mg${}^{2+}$ ions that lowers the repulsive interaction between the monomers.

Although at first glance, the collapse does not seem to be very prominent, it is significant for the length scales of the polyelectrolytes in our system (chain length of only 12 monomers). However, for higher grafting density (Figure 3b), the peak is less distinctive at 3 mM and 50 mM MgCl${}_{2}$ concentrations than 180 mM and nearly plateaus down from 0.8 nm–2 nm. Herein, the high grafting density creates steric repulsion, and the system faces a competition between charge screening and steric repulsion to stabilize the polyelectrolyte structures. At the low MgCl${}_{2}$ salt concentrations (3 mM and 50 mM), the system energetically favors chain stretching to accommodate both charge repulsion and steric hindrance. However, when we further increase the MgCl${}_{2}$ salt concentration to 180 mM, charge screening by the Mg${}^{2+}$ ion dominates over the steric repulsion, and we get a distinct chain collapse near the grafting surface. Figure 3c corresponds to the volume fraction profiles of the polyelectrolyte chains at high grafting density (0.5 chains/nm${}^{2}$) and shows a clearly opposite picture than Figure 3a,b, with the chains stretching, while we increase the salt concentration. In this case, at 3 mM MgCl${}_{2}$, the number of the bound Mg${}^{2+}$ ions is sufficient for mitigating the charge repulsion inside the brush. A further increase in the salt concentration induces additional steric hindrance due to the volume exclusion, and the chains pay in conformational entropy to stretch the chains and accommodate more Mg${}^{2+}$ ions inside the brush. This phenomenon of polyelectrolyte chain stretching with increasing counterion concentration is well known as the “re-entrant phenomena” and has been experimentally verified by several studies [41,42].

**Figure 4:**
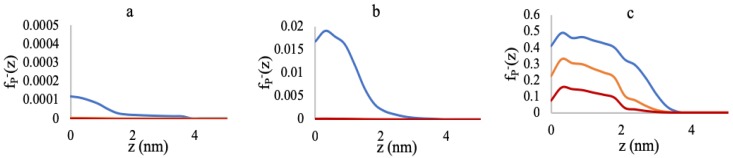
Deprotonated polymer fraction profiles at 3 mM (blue lines), 50 mM (yellow lines), and 180 mM (red lines) MgCl${}_{2}$ concentrations. (**a**) 0.005 chains/nm${}^{2}$, (**b**) 0.05 chains/nm${}^{2}$, and (**c**) 0.5 chains/nm${}^{2}$.

Figure 4 represents the profiles of the deprotonated (negatively-charged) polymer fraction ($f_{P^{-}}\left(z\right)$). This quantity is calculated from the individual monomer-species fractions by,

(33) $$f_{P^{-}}\left(z\right)=\frac{f_{A^{-}}\left(z\right)\langle \phi_{A}\left(z\right)\rangle +f_{G^{-}}\left(z\right)\langle \phi_{G}\left(z\right)\rangle }{\langle \phi_{p}\left(z\right)\rangle }$$

In Figure 4a, at the 0.005 chains/nm${}^{2}$ grafting density, almost 100% of the polymers are bound to Mg${}^{2+}$ for 50 mM and 180 mM salts, and even for 3 mM salt, a very small fraction is deprotonated ($f_{P^{-}}\left(z\right)∼0.0001$), with the rest of the polymers being Mg${}^{2+}$-bound. At 0.05 chains/nm${}^{2}$ (Figure 4b), the polymers are still (nearly) entirely bound to Mg${}^{2+}$ at 50 mM and 180 mM salt concentrations. However, for 3 mM salt, the fraction of the negatively-charged polymers slightly increases.

As we further increase the grafting density up to 0.5 chains/nm${}^{2}$ in Figure 4c, the ionic strength shows a more prominent effect on the chain structure and chemistry. Now, at 3 mM MgCl${}_{2}$, about 50% of the polymers are negatively charged. The increase of the ionic strength to 50 mM and 180 mM decreases the negative charge by binding to more Mg${}^{2+}$ to reduce the negative charge inside the brush.

**Figure 5:**
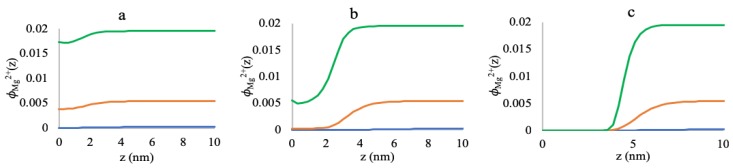
Free Mg${}^{2+}$ volume fraction profiles as a function of distance from the grafting surface at (**a**) 0.005 chains/nm${}^{2}$, (**b**) 0.05 chains/nm${}^{2}$, and (**c**) 0.5 chains/nm${}^{2}$ grafting densities for 3 mM (blue lines), 50 mM (Yellow lines), and 180 mM (green lines) MgCl${}_{2}$ concentrations.

The “re-entrant behavior” shown in Figure 3c can be further explained using Figure 4c and Figure 5. At 3 mM MgCl${}_{2}$, about half of the polymers are negatively charged, and another half is positively charged due to the formation of $AMg^{+}$ and $GMg^{+}$ complexes with the monomers. Hence, the brush environment is nearly neutralized with low or no residual positive or negative charges, and the brush becomes collapsed for that grafting density. Therefore, increasing the salt concentration does not contribute to the global charge neutralization inside the brush; rather, the availability of more Mg${}^{2+}$ ions, that have very high affinity towards binding to the negatively-charged monomers, creates high steric hindrance. As a result, we see stretching of the chains, and the system pays in conformational entropy to reduce steric repulsion inside the brush.

Figure 5 represents the volume fractions of free Mg${}^{2+}$ ions inside the brush and the surrounding medium. At low grafting density (0.005 chains/nm${}^{2}$, Figure 5a), there are significant amounts of free Mg${}^{2+}$ ions inside the brush at all salt concentrations, which is depicted by the small deviation in the free Mg${}^{2+}$ volume fractions from the bulk value.

Here, along with the ion binding, localization of the free Mg${}^{2+}$ ions screens the negative charges inside the brush, which results in a collapse of the polyelectrolyte chains (referring to Figure 3a). However, as we increase the grafting density to 0.05 chains/nm${}^{2}$ (Figure 5b), the amount of the free Mg${}^{2+}$ ions inside the brush drops noticeably from the bulk value, compared to the low grafting density brush. However, the system still entropically favors localization of free Mg${}^{2+}$ ions while staying at a collapsed state (Figure 3b). A further increase in the grafting density up to 0.5 chains/nm${}^{2}$ gives rise to the above-mentioned re-entrant phenomena (Figure 3c). At this point, the brush region acts like a barrier to the free Mg${}^{2+}$ ions, and the brush is completely devoid of the free Mg${}^{2+}$ ions at all the salt concentrations (Figure 5c). For a grafting density this high, the brush is so dense that the binding to the Mg${}^{2+}$ ions increases the volume of the monomers, which also increases the steric hindrance, even at a low MgCl${}_{2}$ salt concentration. Increasing the MgCl${}_{2}$ salt concentration promotes binding to more Mg${}^{2+}$ ions, which is energetically more favorable than bringing the free ions from the bulk and localizing them inside the brush. Hence, the system pays in conformational entropy to stretch the chains to avoid high steric repulsion in the brush, and the re-entrant phenomena arises.

**Figure 6:**
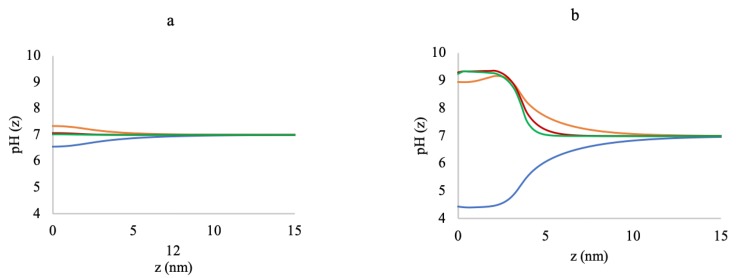
pH profiles along the distance from the grafting surface at (**a**) 0.005 chains/nm${}^{2}$ and (**b**) 0.5 chains/nm${}^{2}$ for 0 mM (blue lines), 3 mM (yellow lines), 50 mM (red lines), and 180 mM (green lines) MgCl${}_{2}$ concentrations.

**Figure 7:**
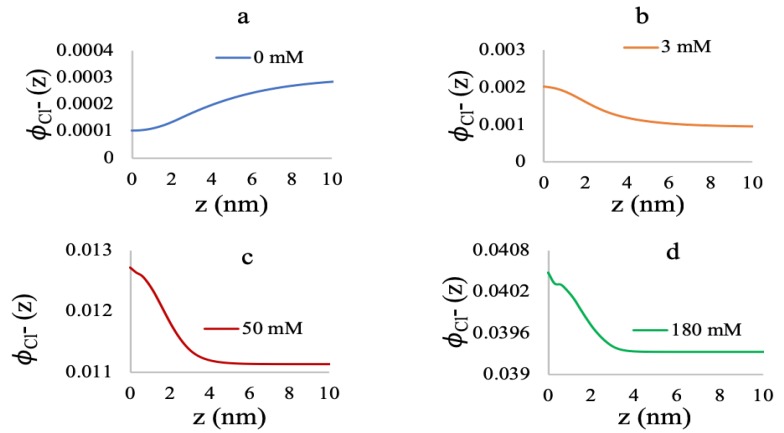
Chloride volume fractions at 0.005 chains/nm${}^{2}$ for 0 mM (**a**), 3 mM (**b**), 50 mM (**c**), and 180 mM (**d**) MgCl${}_{2}$ concentrations.

**Figure 8:**
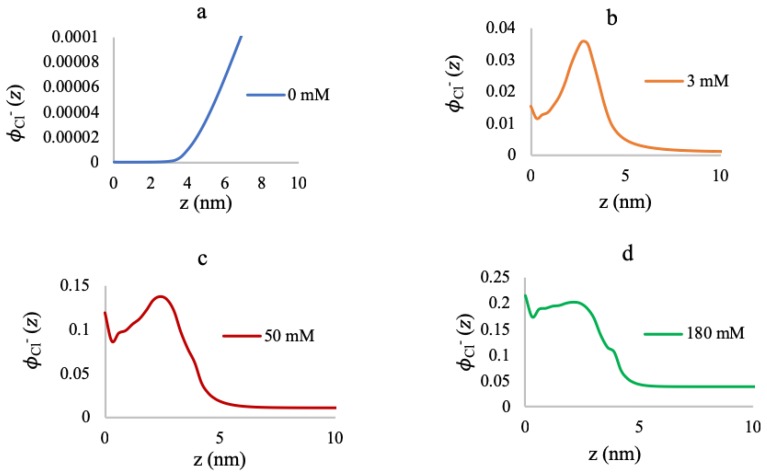
Chloride volume fractions at 0.5 chains/nm${}^{2}$ for 0 mM (**a**), 3 mM (**b**), 50 mM (**c**), and 180 mM (**d**) MgCl${}_{2}$ concentrations.

Figure 6a,b presents the change in local pH inside the brush along the distance from the grafting surface for low (0.005 chains/nm${}^{2}$) and high (0.5 chains/nm${}^{2}$) grafting densities, respectively, for varying ionic strengths. The pH profiles, when no MgCl${}_{2}$ was added in the system, are included in the figures to compare the system’s response with the added MgCl${}_{2}$ salt. At no added MgCl${}_{2}$ (0 mM), the system only had 10 mM NaCl as a background electrolyte to modulate charge regulation inside the brush. At the low grafting density, the pH inside the brush (Figure 6a) only deviates by ∼0.5 from the bulk. However, for the high grafting density, the available Na${}^{+}$ ions are not enough to reduce the negative charges on the polyelectrolytes, and the local pH is much lower with the lowest value of ∼4.5 (Figure 6b). When we add MgCl${}_{2}$ to the system, even as low as 3 mM, the negatively-charged monomers readily bind to the Mg${}^{2+}$ to form $AMg^{+}$ and $GMg^{+}$ complexes that are positively charged. The production of the positively-charged complexes creates a significant change in the pH inside the brush for all the grafting densities. For the low grafting density (Figure 6a), at 3 mM MgCl${}_{2}$, the pH change is only for one unit. As the MgCl${}_{2}$ concentration is increased, the pH surge subsides, and at 180 mM, the charge of the system is almost neutral. However, for a high grafting density (Figure 6b), the addition of only 3 mM MgCl${}_{2}$ salt creates a pH increase of more than four units (from pH = 4.5 to pH = 9.0). As the MgCl${}_{2}$ content is increased, unlike the lower grafting density case, the pH further increases up to ∼9.5. This dramatic change in the local pH with the increase in MgCl${}_{2}$ is consistent with the change in the free chloride volume fraction profiles in Figure 7 and Figure 8.

In Figure 7, volume fractions of free chloride ions along the distance from the grafting surface are reported at a low grafting density of the brush for varying MgCl${}_{2}$ concentrations. When there is no MgCl${}_{2}$ in the system (Figure 7a), the free chloride ions from the dissociation of NaCl salt are excluded from the system and reside in the bulk, due to the repulsion of highly negatively-charged monomers inside the brush. However, when the MgCl${}_{2}$ is added, the positively-charged monomer-cation complex takes the place of the deprotonated monomers. At this stage, the free chloride ions contribute as counterions to minimize the repulsion due to the positively-charged species inside the brush. At 50 mM and 180 mM MgCl${}_{2}$ (Figure 7c,d, respectively), the free chloride ion concentration inside the brush is significantly higher than the bulk, and the local pH is close to neutral. However, for the MgCl${}_{2}$ salt concentration as low as 3 mM, the available chloride ions are not enough to neutralize the positive charge in the brush, and the pH is highest for the specific grafting density.

Figure 8 presents the free chloride volume fractions inside the chain as a function of distance from the grafting surface at high grafting density (0.5 chains/nm${}^{2}$). In the absence of MgCl${}_{2}$, almost all the chloride ions are excluded from the system due to the repulsion by the highly negatively-charged chains, and the counterions (Na${}^{+}$) are excluded due to the high steric hindrance of the crowded brush. As we add MgCl${}_{2}$ salt in the system, the formation of the positively-charged complex now gives rise to electrostatic repulsion inside the brush. It would be thermodynamically favorable for the system to employ negatively-charged chloride counterions to mitigate the electrostatic repulsion. However, the system is already highly dense with the added Mg${}^{2+}$ ions bound to the polymers, and further accommodation of any more species creates high steric hindrance. Hence, the system prefers to stay in a positively-charged condition inside the brush, with a maximum pH of 10.0, and the free chloride ions mostly reside in the outer periphery of the brush, with high volume fractions near the bulk (Figure 8b–d).

**Figure 9:**
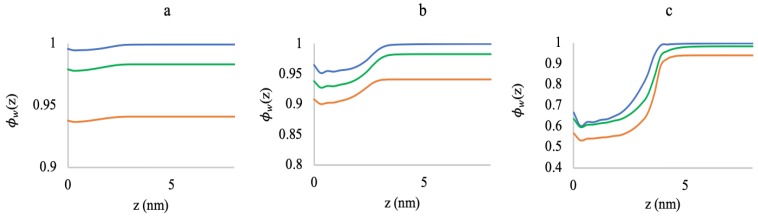
Water volume fraction profiles as a function of distance from the grafting surface at (**a**) 0.005 chains/nm${}^{2}$, (**b**) 0.05 chains/nm${}^{2}$, and (**c**) 0.5 chains/nm${}^{2}$ grafting densities for 0 mM (blue lines), 50 mM (green lines), and 180 mM (yellow lines) MgCl${}_{2}$ salt concentrations. Note the different scales of the plots (a,b,c).

Figure 9 presents the water volume fraction profiles around the oligomer chains at different grafting densities and varying MgCl${}_{2}$ salt concentrations. These plots depict the water displacement around the oligomer chains due to the Mg${}^{2+}$ ion binding with increasing MgCl${}_{2}$ salt concentration. In Figure 9a, at low grafting density (0.005 chains/nm${}^{2}$) and at all the MgCl${}_{2}$ concentrations, the water volume fractions are nearly uniform throughout the polymer layer and the bulk, with a small amount of water displacement near the grafting surface. The water displacement is higher at the high salt concentrations (50 mM and 180 mM MgCl${}_{2}$) compared to when no MgCl${}_{2}$ salt is added. At a medium grafting density (0.05 chains/nm${}^{2}$), the water volume fractions inside the polymer chains varies significantly compared to the bulk at all of the concentrations of the MgCl${}_{2}$ salt. Herein, to accommodate more Mg${}^{2+}$ ions inside the brush, more water is being displaced. Similar to Figure 9a,b, Figure 9c shows that more water is displaced with increasing MgCl${}_{2}$ salt concentration at a high grafting density (0.5 chains/nm${}^{2}$), and the difference in the water volume fraction is very high between the bulk and inside the chain. The high grafting density of the oligomer chains creates high steric repulsion inside the brush that lowers the water content around the oligomers.

**Figure 10:**
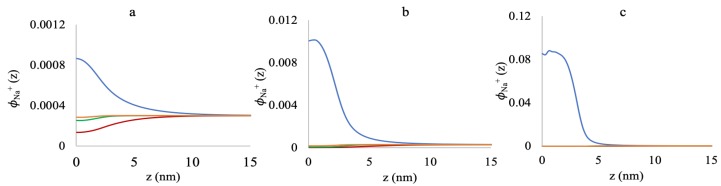
Volume fraction profiles of free Na${}^{+}$ ions as a function of distance from the grafting surface in the presence of 10 mM NaCl salt and at (**a**) 0.005 chains/nm${}^{2}$, (**b**) 0.05 chains/nm${}^{2}$, and (**c**) 0.5 chains/nm${}^{2}$ grafting densities for 0 mM (blue lines), 3 mM (red lines) 50 mM (green lines), and 180 mM (yellow lines) MgCl${}_{2}$ salt concentrations. Note the different scales of the plots (a,b,c).

Figure 10 depicts the volume fraction profiles of free Na${}^{+}$ ions as a function of distance from the grafting surface at varying grafting densities with the presence of varying concentrations of MgCl${}_{2}$ salt. When there is no MgCl${}_{2}$ salt present (the blue lines in the plots in Figure 10a–c), the NaCl salt in the system solely contributes to the charge neutralization inside the oligomer brushes, and hence, the volume fractions of the Na${}^{+}$ ions in the brush layer are very high compared to the bulk. However, as we add the MgCl${}_{2}$ salt in our system, the divalent Mg${}^{2+}$ ions displace the Na${}^{+}$ ions, and we see a significant reduction in the volume fractions of the Na${}^{+}$ ions inside the brush at all the grafting densities and all the concentrations of the MgCl${}_{2}$ salt. At the low grafting density (Figure 10a), we still see a considerable amount of the Na${}^{+}$ ions inside the chain region, due to low steric repulsion inside the brush. Nevertheless, for higher grafting densities of the chains (Figure 10b,c), almost all the Na${}^{+}$ ions are replaced by the divalent Mg${}^{2+}$ cations at all the MgCl${}_{2}$ salt concentrations. These results theoretically support the hypothesis from the relevant experimental study by Holland et al. [34], which states that Mg${}^{2+}$ displaces Na${}^{+}$ through a possible binding pathway with surface-grafted ssDNA oligomers.

In summary, the results in this section portray the complex and coupled interplay between the grafting density of the chains and the ionic strength to govern the structure, ion binding, and local environment inside the brush. The system generates the thermodynamic equilibrium state by adjusting between the conformational entropy, electrostatic potential, and repulsive interactions.

It is important to note that this work considers the Mg${}^{2+}$ ion explicitly, without taking into account the solvation effect. Mg${}^{2+}$ has a strong hydration shell compared to the bulk and binds to the nucleotides via a solvent-mediated-ion pairing [43]. However, to capture the thermodynamics of Mg${}^{2+}$-nucleotide interactions accurately, it is necessary to treat Mg${}^{2+}$ explicitly [26]. This work provides a basis for further theoretical study involving the Mg${}^{2+}$-solvation effect on the tethered ssDNA-Mg${}^{2+}$ binding. Furthermore, this work does not include the possibility of secondary or tertiary structure formation for these particular sequences of nucleic acid oligomers, as no structure formation is evidenced by a relevant experimental study [33]. However, the possibility of secondary or tertiary structure formation will be included in our future studies of these polyelectrolyte systems.

## 4. Conclusions

Divalent metal ion binding to surface-grafted nucleic acid oligomers is investigated by studying the effects of the ionic strength and grafting density on the oligomer structure and chemistry, with a field theoretic molecular model. The cation binding is explicitly included in the model by utilizing the experimentally-derived binding free energies of the relevant reactions. Quantitative assessment of the ion cloud around the oligomers shows a uniform distribution of the ions around different sequences and reinforces the dominance of non-specific electrostatic attraction between the nucleobases and the cations as the driving force for the cation binding [33,44]. The analysis of the system with the variation in the ionic strength and polymer grafting density shows a complex coupling between the chain conformation and the ion cloud to maintain the stability of the system by achieving the minimum energy state. At low grafting density, when the polymers are sparsely grafted, cation binding and ion condensation around the charged oligomers leads to charge neutralization inside the brush, which is accompanied by a chain collapse. At high grafting density, however, cation binding results in the reversal of the oligomer charge, which can no longer be neutralized by the anions due to anion exclusion from the brush to avoid steric repulsion, and hence, we get a highly-stretched polymer brush. Our results also show that the ionic strength has a more prominent effect on the structure and properties of the oligomer brushes when they are densely grafted, compared to their sparsely-grafted counterpart. In its current state, this model can serve as a foundation for field theoretic studies of more complex systems to dissect the ion binding scenario around aptamers and single-stranded nucleic acids. Although, originally constructed to mimic surface-anchored nucleic acid aptamers for the robust design of aptamer-based biosensors and therapeutics, this molecular model can also be employed to understand the molecular level interactions of other natural or synthetic polyelectrolytes with metal ions in a solution environment, for applications ranging from colloid chemistry to drug design for controlled release.
